# The Moss Biomonitoring Method and Neutron Activation Analysis in Assessing Pollution by Trace Elements in Selected Polish National Parks

**DOI:** 10.1007/s00244-020-00755-6

**Published:** 2020-09-08

**Authors:** Grzegorz Kosior, Marina Frontasyeva, Zbigniew Ziembik, Inga Zincovscaia, Agnieszka Dołhańczuk-Śródka, Barbara Godzik

**Affiliations:** 1 Institute of Environmental Engineering and Biotechnology, University of Opole, ul. kard. B. Kominka 6, 45-032 Opole, Poland; 2 Frank Laboratory of Neutron Physics, Division of Nuclear Physics, Department of Neutron Activation Analysis and Applied Research, Joint Institute for Nuclear Research, Moscow Region, Russian Federation; 3 Horia Hulubei National Institute for R&D in Physics and Nuclear Engineering, Bucharest, Magurele, Romania; 4 W. Szafer Institute of Botany, Polish Academy of Sciences, Kraków, Poland

## Abstract

The concentrations of trace elements in feather moss *Pleurozium schreberi* (Brid.) Mitt. were used to indicate the relative levels of air pollution by trace elements in Polish national parks. *Pleurozium schreberi* was collected from nine national parks. The highest concentrations were recorded in the moss samples from the southern and most industrialised part of the country; the lowest from northern and north-eastern Poland. A comparison of data obtained from Polish national parks in the 1970s and 1990s showed a significant decrease in the concentrations of heavy metals. In the linear covariability estimation, the *t* quantile approach was used for multi-element comparison. A number of positive covariabilities were observed. This is a result of anthropogenic activity and the geochemical characteristics of the local environment, including crust composition to which soil composition is related. The statistical approach of *t* quantile to study common relationships between element concentrations can be used in the interpretation of biomonitoring research results in similar studies.

**Electronic supplementary material:**

The online version of this article (10.1007/s00244-020-00755-6) contains supplementary material, which is available to authorized users.

In Poland, a national park is a protected area because of its advantages; mainly its outstanding natural value for environmental, scientific, social, cultural, and educational reasons. The national parks are one of the Poland’s strategic natural resources. Therefore, monitoring of the contamination degree of national parks is very important. Industrial and agricultural activities in national parks are prohibited, but airborne chemicals can travel long distances from their sources and therefore can affect ecosystems over broad spatial scales and at locations far from the emission sources (Samecka-Cymerman et al. 2012). Trace elements are emitted by various combustion sources, which can undergo chemical reactions in the atmosphere and fall to the earth as wet deposition (rain, snow), occult deposition (cloud, fog), or dry deposition (dry particles, gas) (Radaman 2004; Schröder et al. 2010). After atmospheric deposition, toxic metals can accumulate in the environmental compartments and tissues of living organisms, increasing the risk of harmful effects (Livett 1988; Seinfeld and Pandis 2006). National parks, which are considered to be the most natural and least contaminated areas, also are affected (Grodzińska 1978).

The problem of pollution by trace elements is still unsolved, and in some cases growing. Therefore, there is a need to continue deposition analysis of these pollutants in the environment (Kosior et al. 2010). Today, moss biomonitoring is a part of pollution monitoring programmes in most European countries as it gives evidence of the anthropogenic impact in urban areas due to vehicular traffic and fossil fuel combustion. It also identifies other sources of heavy metal pollution, such as ore exploitation, agricultural activities, etc. (Aničić 2006; Barandovski et al. 2008; Frontasyeva et al. 2016). Results from moss surveys allow examination of both spatial and temporal trends in metal concentration/deposition, and the identification of areas exposed to the high deposition of metals from long-range transport and local sources. Mosses have a high interception potential for particulate material and precipitation and, therefore, contain significantly higher trace element concentrations than vascular plants and fungi (Ugur et al. 2003; Skuterud et al. 2005). Due to their continuous accumulation of elements, mosses offer information about the sources of pollution long after the pollution episode itself took place (Golubev et al. 2005).

We chose the terrestrial carpet-forming, ectohydric moss *Pleurozium schreberi* for this study. Ectohydric mosses obtain most trace elements and nutrients directly from the atmosphere (wet and dry deposition) with little uptake from the substrate and therefore are suitable for monitoring airborne pollutants. This species has been demonstrated to be a suitable bioindicator of inorganic substances originating from the atmosphere and has been widely used to map and monitor airborne pollution in European countries (Halleraker et al. 1998). Due to the lack of root systems, mosses depend largely on atmospheric depositions for their nutrient supply and have a great capacity to retain many elements. Mosses lack a cuticle, or it is very thin, due to which their leaves are highly permeable to ions of trace elements, and they usually have a high surface-to-volume ratio (5–10 times higher than vascular plants) (Tyler 1990). Element concentrations as measured in moss samples are supposed to represent the accumulated load of the past 2–3 years (Pakarinen and Rinne 1979). Due to this accumulation, element concentrations measured in moss are much higher than in other sample materials, such as precipitation, dust, or other plants, and thus are easier to measure (Asakawa et al. 2013).

The main objective of the present study was to test the statistical approach of the *t* quantile to compare elements bioaccumulated in *P. schreberi* collected from Polish national parks (PNP) and to compare the results between lowland and highland/mountain parks. Our study also examined the view that special care should be taken in regional comparisons of bryophyte concentrations of trace elements. A minor objective is to compare the results with earlier data from Poland. Novel statistical analysis techniques have been used to evaluate the results.

## Materials and Methods

### Sampling Design

**Fig. 1:**
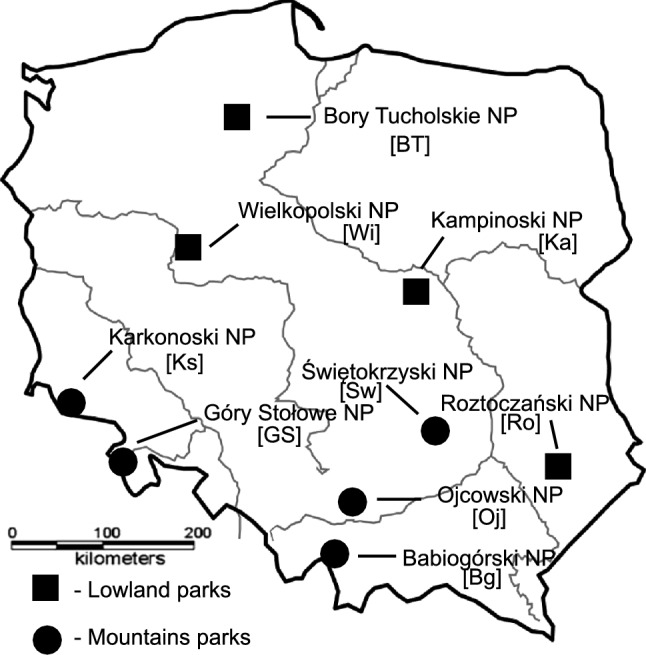
Localisation of the studied national parks. Samples of *P. schreberi* moss were collected from four lowland parks: Bory Tucholskie [BT], Wielkopolski [Wi], Kampinoski [Ka], Roztoczański [Ro] and from five upland and mountainous parks: Karkonoski [Ks], Gór Stołowych [GS], Ojcowski [Oj] Babiogórski [Bg] and Świętokrzyski [Sw]

Samples of *P. schreberi* moss were collected from four lowland parks: Bory Tucholskie NP, Wielkopolski NP, Kampinoski NP, Roztoczański NP, and from five highland/mountain parks: Karkonoski NP, Gór Stołowych NP, Ojcowski NP, Babiogórski NP, and Świętokrzyski NP (Fig. 1). In the figures, these names are abbreviated to BT, Wi, Ka, Ro, Ks, GS, Oj, Bg, and Sw, respectively. Mosses were collected from an area measuring about 50 m × 50 m and at least 5 m away from the canopy of the trees so as not to be directly exposed to through fall precipitation. Within the central part of each of the selected site, three sub-squares of 2 × 2 m, covered with *P. schreberi*, were selected randomly for the collection of this native moss (only the green parts of moss were taken). Each plant sample consists of a mixture of three subsamples. The design of the moss samples sites was performed according to the protocol adopted within “Heavy Metals in European Mosses: 2000/2001 Survey” (UNECE ICP Vegetation 2011). Sampling and handling was performed using polyethylene gloves and bags. After selection and removing foreign bodies, the moss samples were transported to the laboratory. In the laboratory, the samples were carefully cleaned of all dead material and attached litter.

### Laboratory Analyses

#### Instrumental Neutron Activation Analysis

The concentration of elements in the *P. schreberi* moss samples was determined by a multielement instrumental neutron activation analysis (NAA) at the IBR-2 reactor, FLNP JINR, and Dubna. The characteristics of neutron flux density in the two irradiation channels equipped with the pneumatic system and the registration of gamma spectra can be found elsewhere (Frontasyeva 2011). To determine the concentration of elements with short lived isotopes (Al, Ca, Cl, I, Mg, Mn, and V), samples were irradiated for 3 min and measured for 20 min. To determine the concentration of elements with long lived isotopes (Na, Sc, Cr, Fe, Co, Ni, Zn, As, Se, Rb, Sr, Zr, Mo, Sb, Cs, Ba, La, Ce, Sm, Eu, Tb, Hf, Ta, W, Th, and U) (Table 1), the cadmium-screened channel 1 was used. Samples were irradiated for 4 days, repacked, and measured twice using HP germanium detectors after 4 and 20 days of decay, respectively. The NAA data processing and the determination of element concentrations were performed using software developed in FLNP JINR (Dmitriev and Yu 2013).

**Table 1:** Radionuclides used for neutron activation analysis and their γ-energies

Element	Isotope	Half life	Gamma peak (keV)	Method of analysis*
Al	^28^Al	2.2 m	1778.9	2
As	^76^As	26.3 h	559.1	3
Ba	^131^Ba	11.8 d	496.8	4
Br	^82^Br	35.3 h	776.5	3
Ca	^49^Ca	8.7 m	3084.4	2
Ce	^141^Ce	32.5 d	145.4	4
Cl	^38^Cl	7.7 m	1881.2	2
Co	^60^Co	5.3 y	1173.1	4
Cr	^51^Cr	27.7 d	320.1	4
Cs	^134^Cs	2.1 y	795.8	4
Fe	^59^Fe	44.5 d	1099.2	4
Hf	^181^Hf	42.4 d	482	4
K	^42^ K	12.4 h	1524.7	3
La	^140^La	40.2 h	1596.5	3
Mg	^27^ Mg	9.5 m	1014.1	2
Mn	^56^Mn	2.6 h	1810.7	2
Mo	^99^Mo	66 h	140.5	3
Na	^24^Na	14.7 h	2753.6	3
Rb	^86^Rb	18.7 d	1076.6	4
Sb	^124^Sb	60.2 d	1691	4
Sc	^46^Sc	83.8 d	889.2	4
Sr	^85^Sr	64.8 d	514	4
Th	^233^ Pa	27 d	312	4
U	^239^Np	2.4 d	228.2	3
V	^52^ V	3.8 m	1434.1	1
Zn	^65^Zn	244 d	1116	4

^*^ Method 1: conventional NAA, measured after 2–3 min of decay

Method 2: conventional NAA, measured after 9–10 min of decay

Method 3: epithermal NAA, measured after 4–5 days of decay

Method 4: epithermal NAA, measured after 20–23 days of decay

#### Quality Control for Instrumental Neutron Activation Analysis

To evaluate the precision and accuracy of the results, the standard reference materials (SRMs) [1572 (citrus leaves), 2710 (Montana soil), 1632c (trace elements in coal), and 1547 (pPeach leaves)] were simultaneously irradiated with the analysed samples. The results obtained for the SRMs, as well as their certified values, are shown in Table A—Supplementary Information. The measured concentrations and certified values are in good agreement, with the overall precision for measurement between 1 and 10% for most of the elements, except Mo and Sr, which were found to have a higher uncertainty.

#### Atomic Absorption Spectrophotometry Analysis

The total content of Cd, Cu, and Pb was determined by the ASS method. The unwashed samples were air-dried at 30 °C. To conduct the determination of the elements, the samples were digested with 3 ml of nitric acid (ultra-pure, 65%) and 2 ml of perchloric acid (ultra-pure, 70%) in a CEM Mars 5 microwave oven (Matusiewicz 2003). The plant digests were analysed for Cd, Cu, and Pb using an ETAAS with a Graphite Furnace GF3000, both Atomic Absorption Spectrophotometry AVANTA PM GBC Scientific Equipment (Lajunen and Peramaki 2004). All elements are determined against standards (Atomic Absorption Standard Solution from Sigma Chemical Co.) and blanks containing the same matrix as the samples and subjected to the same procedure. All results for the plants are calculated on a dry weight basis. The accuracy of the methods applied for the determination of elements after microwave-assisted digestion of plants and soil samples is checked by analysing certified reference materials. We used bush branches and leaves from DC73348 LGC standards and RTH 907 Dutch Anthropogenic Soil (Wageningen Evaluating Programmes for Analytical Laboratories, WEPAL) as certified reference materials (Table B—Supplementary Information). The coefficient of variance (CV) is calculated for the determined concentrations of elements in the reference materials.

### Statistical Analysis

Because concentration represents the amount of a selected substance in the total amount of all components, its analysis requires application of appropriate mathematical methods (Aitchison 2003; Pawlowsky-Glahn and Buccianti 2011). The sample space of our measurement results is a set of compositions. Composition of a system composed of *D* components can be calculated by the closure operation C, defined as follows:

1 $$C({\mathbf{z}}) = \left[ {\frac{{k_{s} {\mathbf{z}}_{1} }}{{\sum\nolimits_{j = 1}^{D} {{\mathbf{z}}_{j} } }},\frac{{k_{s} {\mathbf{z}}_{2} }}{{\sum\nolimits_{j = 1}^{D} {{\mathbf{z}}_{j} } }}, \ldots ,\frac{{k_{s} {\mathbf{z}}_{D} }}{{\sum\nolimits_{j = 1}^{D} {{\mathbf{z}}_{j} } }} = {\mathbf{x}}} \right]$$

where **z** is the vector of component amounts (mass or count, for example), *k* depends on the units of measurement and **x** is the vector of the compositions. For mg/kg, the *k* value is 10^6^. The subcomposition can be obtained by applying the closure operation to a vector of *S* parts taken from **z** (*S* < *D*).

To describe the difference between two compositions, the scalar measure of distance was introduced. The Aitchison distance *d*_A_ between two compositions **x**[x_1_,x_2_,.,x_D_] and **y**[y_1_,y_2_,.,y_D_] is described by the formula (Pawlowsky-Glahn and Buccianti 2011):

2 $$d_{{\text{A}}} ({\mathbf{x}},{\mathbf{y}}) = \sqrt {\sum\limits_{i = 1}^{D} {\left( {\ln \frac{{x_{i} }}{{g({\mathbf{x}})}} - \ln \frac{{y_{i} }}{{g({\mathbf{y}})}}} \right)}^{2} }$$

An insight into the data structure can be delivered by cluster analysis methods. Clustering comprises unsupervised methods that can be used to organise data into groups based on similarities among the individual data items. Clustering involves dividing data points into clusters so that items in the same class are as similar as possible. Different types of similarity measures may be used to identify classes; in this work, the distance between compositional points in simplex space, defined in Eq. 2, was used. In hard clustering (non-fuzzy), data are divided into crisp clusters, where each data point belongs to exactly one cluster. In fuzzy clustering, the data points can be assigned to more than one cluster. For each of the points, its membership grade is determined to indicate the degree to which the data points belong to the different clusters. Initial results, assuming the number of clusters, were used in fuzzy clustering analysis.

For each sampling point, the contribution of a cluster was calculated. To estimate the contribution of the *k*th element in the *j*th cluster structure, the value of the following expression can be considered:

3 $$FC_{j} = \sum\limits_{i = 1}^{n} {f_{ji} C_{ki} }$$

A high value of the chemical element concentration and its high contribution in the cluster produces big values of the *FC*_j_ vector elements. Low concentrations and contributions produce low values of the vector’s elements. A big *FC*_jk_ vector element assume the significant contribution of the *k*th element in the *j*th cluster structure.

The compositional data are not independent of each other; if content of one of the components increases, the others have to decrease. It is the reason why the results of standard statistical analysis of the relationships between raw components or parts are spoiled by spurious effects (Pawlowsky-Glahn and Buccianti 2011; Pawlowsky-Glahn and Egozcue 2006). The particular properties of compositional data preclude the application of standard statistical techniques on such data in its raw form. For example, variances of constrained, compositional data components are not independent. Variance in a component concentration may not be strictly related to variance in the component abundance, because this variability also is affected by variabilities in the abundances of the other components. As the result of the closure operation, variance of a component and its covariances with the other components are bound by the relationship:

4 $${\text{cov}} ({\mathbf{x}}_{1} ,{\mathbf{x}}_{2} ) + \cdots + {\text{cov}} ({\mathbf{x}}_{1} ,{\mathbf{x}}_{D} ) = - {\text{var}} ({\mathbf{x}}_{1} )$$

The application of classical data analysis methods for the statistical analysis of compositional data may lead to false or delusive conclusions. This is why in data interpretation statistical methods designed for compositional data elaboration were used. Particularly, the utilisation of a classical correlation estimator in the statistical analysis of compositional data can lead to delusive conclusions (Filzmoser and Karel 2008). An alternative method, appropriate for covariability estimation in compositional data, is based on the variation matrix **T**. The elements *t* of this matrix are variances of the concentration pairs’ log-ratios (Aitchison 2003):

5 $$t_{jl} = {\text{var}} \left( {\ln \frac{{x_{j} }}{{y_{l} }}} \right),j,\,l = 1, \ldots ,D$$

The variance of the log-ratio is small when changes in both concentrations follow the same trend, i.e., both values simultaneously increase or decrease. The biggest log-ratio variance is observed when concentrations follow opposite trends. An increase in one variable’s values accompanied by a decrease in the values of the second one produces highly differentiated log-ratios. As the result, the biggest *t* value is observed. To distinguish between these two types of covariability, the terms “positive” (an increase in both concentrations) and “negative” (an increase in one concentration and a decrease in the second) are used. However, interpretation of a high *t* is restricted. The actual relationship between abundances of components in a system cannot be reflected by the corresponding *t* value.

The *t* values between these characteristics for negative or positive covariabilities describe a random or non-linear relationship between the concentrations. The intermediate values of randomly arranged data can be used in the assessment of the actual *t* classification.

The distribution function of *t* can be estimated on the basis of the actual data. Data permutation in the first variable, keeping an unchanged sequence in the second one, produces a number of *t* values. Most of them correspond to a random arrangement of pairs of variables. A fraction of the lowest *t* values correspond to concentrations positively related with each other. A fraction of the highest *t* values correspond to negative covariability between concentrations (Ziembik and Dołhańczuk-Śródka 2015). In this work, the *t* distribution quantile *q*_0.01_ was the upper limit for positive covariability, and *q*_0.99_ was the lower limit for a negative one. Although, as mentioned earlier, Pearson’s correlation coefficient *r* is generally not appropriate for covariability assessment among concentrations, its value was calculated to compare conclusions with the ones related to the *t* estimates.

In computations, the R language (R Development Core Team 2015) and functions from the libraries, “cluster” (Kaufman and Rousseeuw 2005; Maechler et al. 2014) and “compositions” (van den Boogaart et al. 2014) were used.

## Results and Discussion

The calculated statistical parameters of the data are presented in Table 2 and Table D—Supplementary Information. The following parameters are shown: (*min*—minimal value, *q*_1_—lower quartile, median, arithmetic mean, *q*_3_—upper quartile, *max*—maximal value, SD—standard deviation, and MADN—normalised median absolute deviation about the median (Maronna et al. 2006). The MADN parameter can be regarded as a robust alternative to the SD. If no outliers are in the vector of the normally distributed data then the MADN and SD values are similar to each other. Comparison of the median and mean values of the concentration indicates skewness in the data distribution. The most significant difference between the mean and median is observed for Sc. The mean concentration of this element was twice as high as the median. For Hf, Co, Fe, Na, V, Cs, Al, Mg, and Cr, the mean to median ratio exceeds 1.5. For Sb, the lowest ratio of 1.0 was obtained. A ratio lower than 1.2 was observed for As, Ca, Br, K, Mo, and Zn. For the SD and MADN, similar differences occurred. The biggest SD to MADN ratio, calculated for Sc, was 6.4. Ratios higher than 3.0 were calculated for Co, Cr, Na, Fe, and V concentrations. The lowest ratios, not higher than 1.2, were found for Mn, Rb, Sb, Mo, U, and Br. Comparison of the statistical parameters between mountain parks and lowland parks is presented in Table 2. Concentrations of most elements (As, Ba, Ca, Cd, Cl, Cr, Cu, Mo, Pb, Sr, and Zn) were higher (Wilcox test, *p* < 0.05) in mosses collected from highland and mountainous parks than from lowland parks, with the opposite relationship only for Mn. In the first Polish biomonitoring study with mosses (Grodzińska 1978), most of the highest heavy metal concentrations also were found in *P. schreberi* and *Hylocomium splendens* species in highland and mountain parks (Ojcowski, Świętokrzyski and Babiogórski), whereas the lowest concentrations were recorded in maritime and lowland parks (Woliński, Słowiński, and Białowieski). In the southern part of the country, there are mountainous regions, where the rates of atmospheric element deposition may vary locally which is related, among others things, to altitude, which strongly influences the amount of precipitation, the dry deposition of gases and particles, and the wet deposition of cloud or fog droplets (Gerdol and Bragazza 2006). The pattern obtained in this study confirms the results from other European areas. In the northern and eastern Alps (Austria), mosses have been collected from transects along altitudinal gradients on five mountain ranges (Zechmeister 1995). The results showed a remarkable increase of Pb, Cd, Zn, and S concentrations as the altitude rises. High levels of precipitation were strongly correlated with heavy metal deposition, and this seems to be the main source of heavy metal fallout at higher altitudes. Larger amounts of windblown, indigenous particles also were considered for several heavy metals (e.g., V) higher levels (Zechmeister 1995). In the study from north Italy (Gerdol and Bragazza 2006), concentrations of anthropogenic pollutants (especially Cd and Pb) peaked at mid altitude (1400–1800 m) where the frequency of cloud cover was the highest. The authors concluded that the deposition of trace elements by cloud water, which may account for a significant fraction of the total deposition of anthropogenic trace elements, is closely related to the cloud cover frequency (Gerdol and Bragazza 2006). On the other hand, in southern Poland there are many sources of pollution from local industry, which may be partly responsible for the higher concentrations of elements in mountainous areas (Grodzińska et al. 1999; Grodzińska and Szarek-Łukaszewska 2001). The Upper Silesian Ecological Hazard Area is within the southern Poland. This region has been recognised as area of ecological disaster (Wcisło et al. 2002). Additionally, this area is affected by heavy traffic and airborne transboundary pollution from Germany and the Czech Republic (Appleton et al. 2000). It also was demonstrated that a group of elements may have crustal origin in areas of upland and highland (Agnan et al. 2014; Aničić et al. 2007), which is confirmed in this research by the convariability method described below. The values in this study were compared with the reference values from Markert et al. (2014). *P. schreberi* from both groups of parks were characterised by higher levels of Al, Ce, Cr, and Fe (Table 2). Furthermore, mosses from the highland/mountain parks were characterised by higher levels of Cs, Pb, and Sc. This suggests a significant impact of elements regarded to be of crustal origin on the total content of all studied elements.

**Table 2:** Minimum (min), maximum (max), mean (avg), and standard deviation (SD) of the concentration (mg kg^−1^) of elements in *P. schreberi* from national parks divided into two groups: mountainous parks and lowland parks

	Min	Max	Avg	SD	1975^2^		Min	Max	Avg	SD	Wilcox test	Reference values^1^	1975^2^	Poland 1995^3^
	Mountainous parks						Lowland parks							
Al	215	3770	1085	1128		Al	105	1550	695	519	0.24	90–530		
As	0.1	1	0.4	0.2		As	0.1	0.3	0.2	0.1	0.01	0.01–1.5		
Ba	11	78	30	21		Ba	4	23	12	5	0.00	10–100		
Br	1.1	5.2	2.4	1.2		Br	0.8	3	1.7	0.7	0.06	15–600 mg/L		
Ca	1310	9030	4142	2303		Ca	740	3230	2163	765	0.00	1%		
Cd	0.2	1.1	0.5	0.2		Cd	0.2	0.4	0.3	0.05	0.00	0.03–0.5		0.5
Ce	0.2	3.9	1.3	1.3		Ce	0.3	1.5	0.8	0.4	0.56	0.25–0.55		
Cl	58	813	349	223		Cl	39	340	137	83	0.00	0.2–2%		
Co	0.1	2.3	0.5	0.7		Co	0.1	0.4	0.2	0.1	0.12	0.02–0.5		
Cr	0.5	17	4.2	5.1	4.3	Cr	0.4	3	1.6	0.7	0.04	0.2–1	2.8	1.8
Cs	0.1	2.2	0.5	0.6		Cs	0.04	0.9	0.3	0.2	0.49	0.03–0.44		
Cu	5.2	10.7	8.8	1.7		Cu	4.9	8.1	6.4	1.3	0.00	2–20		10.7
Fe	155	4600	977	1344	1802.9	Fe	119	859	392	246	0.14	5–200	848	448
Hf	0.05	0.86	0.19	0.23		Hf	0.02	0.24	0.11	0.08	0.48	0.001–1		
K	3800	14,900	7816	3028		K	2300	11,800	5887	2343	0.06	0.5–3.4%		
La	0.1	1.9	0.7	0.6		La	0.1	0.9	0.4	0.2	0.22	0.15–0.25		
Mg	510	6320	2395	1869		Mg	454	3780	1489	1054	0.14	1000–10,000		
Mn	28	613	207	179		Mn	140	671	406	185	0.00	1–700		
Mo	0.08	0.41	0.21	0.09		Mo	0.07	0.19	0.13	0.04	0.01	0.03–5		
Na	102	1450	355	405		Na	99	285	166	57	0.22	35–1000		
Pb	5.8	22.3	10.7	5.4	73.4	Pb	3.1	7	4.9	1.4	0.00	0.1–5	29.6	17.3
Rb	7	100	39	31		Rb	6	59	34	15	0.97	1–50		
Sb	0.05	0.31	0.2	0.07		Sb	0.07	0.3	0.17	0.07	0.26	0.1–200		
Sc	0.04	2.4	0.4	0.7		Sc	0.03	0.3	0.1	0.1	0.34	0.01–0.2		
Sr	2.6	34.9	14.2	10		Sr	3.1	11.8	7.4	2.6	0.05	3–400		
Th	0.06	0.44	0.16	0.11		Th	0.03	0.22	0.1	0.06	0.08	0.03–1.3		
U	0.02	0.18	0.06	0.04		U	0.02	0.07	0.04	0.02	0.28	0.005–0.06		
V	0.4	10.1	2.5	3.1		V	0.3	2.8	1.3	0.9	0.18	0.001–10		
Zn	39	83	54	14		Zn	12	62	40	11	0.01	15–150		48

Comparison of the element concentrations in mosses collected from lowland parks with mosses from mountain parks was done using the Wilcox test (results statistically significant in bold *p* < 0.05—probability level).^1^—(Markert et al. 2014^2,3^; Grodzińska et al. 1999)

**Fig. 2:**
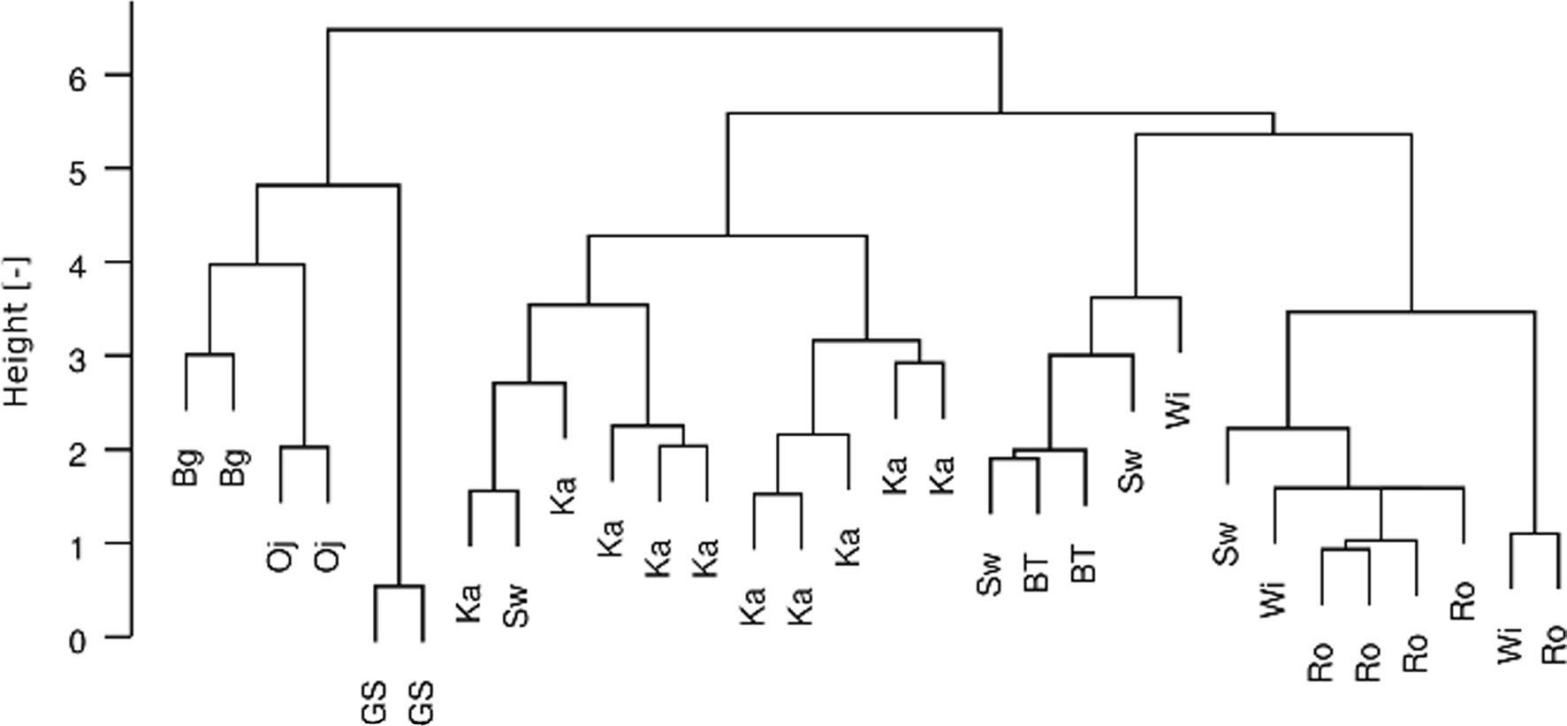
Clustering methods were applied and the results are presented in the tree diagram. The structures of dendrograms constructed using a divisive algorithm are shown

The values were compared to the average concentrations of elements (Cd, Cr, Cu, Fe, Pb, and Zn) for the whole area of Poland. In the case of lowland PNP, we obtained lower values for all elements, with the most significant decreases observed for Cu and especially for Pb (Grodzińska et al. 1999; Table 2). Interestingly, for the mountain parks in this study, we received lower values for Cu and Pb (the most significant decrease being for the latter), the level of Cd was similar for both mountain and lowland parks, whereas for other elements, their concentrations were higher in mountain parks. The concentrations of Cr, Fe, and Pb were lower in comparison with the concentrations for PNP, both mountain and lowland, in 1975 (Grodzińska et al. 1999; Table 2). Again, the most significant decrease was recorded for Pb in both lowland and mountain parks. Since the 1970s, a continuous decrease in the concentrations of heavy metals determined in mosses was observed, which is confirmed by Grodzińska et al. (1999) and by measuring metal emissions by the Central Statistical Office – GUS (GUS 2016) (Table C—Supplementary Information). These authors revealed that the decrease over 20 years (1975–1995) in 12 PNP, which were fairly evenly distributed across Poland, was also evident. Moreover, in the study by Kłos et al. (2015), the analyses of the results of the biomonitoring research carried out during the years 1975–2014 indicate an improvement in the quality of the environment in the studied areas of Poland. The mean concentrations of Ni, Cu, Zn, Cd, and Pb in the samples collected in 2014 in the woodlands of north-eastern Poland were even comparable to the mean concentrations of metals determined in mosses collected in the Svalbard archipelago (N Norway), regarded as relatively clean, during the last three decades. To assess the grouping of trends in the data, clustering methods were applied and the results are presented in a tree diagram (Fig. 2). This is a dendrogram constructed using a divisive algorithm. The branches in the tree are marked with the abbreviations for the names of each national park. A group of branches representing the composition of samples collected in the Babiogórski, Ojcowski, and Góry Stołowe PNP appears in the dendrogram. The composition of the samples from Karkonoski PNP seems to form a cluster, but intrusions of branches representing samples from the other PNPs are observed in its structure. The structure of dendrogram supposes an influence of site localisation on sample composition but it is not critical. Some other, currently uncontrolled, factors significantly influenced the elemental composition of the samples. To look for actually overlapping data structures, the fuzzy clustering method was applied. The algorithm applied in the “fanny” function used in computations produced mostly inconsistent results. Only for the number of clusters = 2 and the membership exponent = 2 were the computation results reasonable. To estimate the influence of an element on the cluster formation, the components of the *FC matrix* were calculated. A significant contribution of an element in the cluster results in a big component value compared with the components representing other clusters. The components of the *FC matrix* calculated using our data were similar. The lowest component ratio was 0.8 for the alkali metals Rb and Cs, and the biggest one was 1.4 for Sc and Fe (crustal origin elements mostly). These values assume, at most, a weak trend to cluster formation in the data. Weak grouping in the data enables application of covariability analysis. To assess the linear covariability between pairs of element concentrations, the quantiles of the *t* parameter distribution were calculated. In Table 3, the results of the computations are shown. Symbols describe the covariability character, i.e., “r” is for random or no linear relationships between concentrations, “p” is for positive covariability and “n” is for negative covariability. For comparison, the commonly used data interpretation Pearson's correlation coefficients r were calculated. The *r* parameters were tested for a 0 value in the population, at a *p*-level of 0.02. For 2D normally distributed data composed of variable pairs representing 30 cases, the critical, absolute |*r*_c_| value is 0.423. In Table 3, positive correlations bigger than *r* are in bold and negative correlations less than *r* are underlined. Linear covariabilites estimated with the *t* quantiles and the correlation coefficient *r* often lead to similar, but not identical, conclusions. For example, both the *t* quantile and *r* for the Na and Mg pair suppose positive covariability in these metals’ concentrations. But though *r* for the Al and Na concentrations is big, the *t* quantile indicates no linear relationship between these variables. In contrast, the poor correlation between Na and K concentrations is associated with the positive covariability supposed by the *t* quantile. In the assessment of the covariabilities in the data, the *t* quantile is preferred due to its compositional coherence. For this reason, this parameter is used in statistical inference, and the *r* parameter influence on the data interpretation is limited.

**Table 3:** Covariabilities in concentration pairs estimated with Pearson's correlation coefficient (above the table diagonal) and the *t* quantile encoded “r” for random or nonlinear, “p” for positive and “n” for negative covariability

	Na	Mg	Al	Cl	K	Ca	Sc	V	Cr	Mn	Fe	Co	Cu	Zn	As	
Na		p	r	p	r	p	p	r	p	r	p	p	p	r	p	Na
Mg	**0.71**		r	p	p	p	r	r	r	r	r	r	p	r	r	Mg
Al	**0.81**	**0.56**		p	r	p	p	p	p	r	p	p	r	r	p	Al
Cl	**0.60**	0.40	**0.45**		p	p	r	p	r	r	p	r	r	r	p	Cl
K	**0.42**	**0.57**	0.21	**0.66**		p	r	r	r	r	r	r	r	r	r	K
Ca	**0.76**	**0.67**	**0.80**	**0.66**	**0.48**		p	p	r	r	p	p	r	r	p	Ca
Sc	**0.95**	**0.68**	**0.90**	**0.48**	0.27	**0.80**		p	p	r	p	p	r	p	p	Sc
V	**0.89**	**0.65**	**0.98**	**0.49**	0.28	**0.84**	**0.96**		p	r	p	p	r	r	p	V
Cr	**0.94**	**0.63**	**0.86**	**0.53**	0.28	**0.76**	**0.97**	**0.93**		r	p	p	r	p	p	Cr
Mn	− 0.27	0.15	− 0.11	− 0.30	0.03	− 0.28	− 0.27	− 0.16	− 0.32		r	r	r	n	r	Mn
Fe	**0.93**	**0.65**	**0.90**	**0.51**	0.26	**0.82**	**0.99**	**0.96**	**0.97**	− 0.30		p	r	p	p	Fe
Co	**0.94**	**0.67**	**0.89**	**0.47**	0.26	**0.80**	**1.00**	**0.96**	**0.98**	− 0.27	**0.99**		r	p	p	Co
Cu	**0.44**	**0.55**	0.06	0.24	**0.42**	0.32	0.30	0.18	0.36	− 0.18	0.27	0.33		r	r	Cu
Zn	**0.49**	0.27	**0.44**	**0.59**	0.24	**0.56**	**0.53**	**0.48**	**0.55**	− 0.43	**0.59**	**0.54**	0.20		p	Zn
As	**0.79**	**0.55**	**0.74**	**0.52**	0.29	**0.79**	**0.81**	**0.79**	**0.80**	− 0.29	**0.85**	**0.85**	0.41	**0.57**		As
Br	0.23	0.05	0.12	0.33	0.12	0.32	0.15	0.15	0.18	− 0.17	0.20	0.22	0.33	0.23	**0.60**	Br
Rb	0.16	− 0.04	− 0.01	0.18	0.24	− 0.12	0.05	0.03	0.16	− 0.09	0.00	0.04	0.22	0.06	− 0.19	Rb
Sr	**0.49**	0.24	0.40	0.27	0.17	**0.56**	**0.47**	**0.46**	**0.46**	− 0.41	**0.47**	**0.52**	**0.44**	0.39	**0.56**	Sr
Mo	**0.69**	0.35	**0.54**	0.39	0.12	**0.62**	**0.69**	**0.61**	**0.72**	− 0.52	**0.70**	**0.72**	**0.48**	0.37	**0.76**	Mo
Cd	− 0.01	0.04	0.08	**0.45**	0.12	**0.45**	0.05	0.06	0.05	− 0.43	0.13	0.06	0.06	**0.45**	0.32	Cd
Sb	0.06	− 0.07	0.31	0.00	− 0.29	0.23	0.24	0.27	0.22	− 0.21	0.33	0.27	− 0.17	**0.45**	**0.50**	Sb
Cs	0.05	− 0.15	− 0.09	0.18	0.16	− 0.09	− 0.04	− 0.04	0.06	− 0.24	− 0.07	− 0.05	0.07	0.03	− 0.10	Cs
Ba	**0.77**	**0.48**	**0.67**	**0.49**	0.29	**0.77**	**0.79**	**0.74**	**0.82**	− 0.48	**0.81**	**0.82**	**0.52**	**0.56**	**0.86**	Ba
La	**0.69**	**0.43**	**0.74**	**0.42**	0.13	**0.71**	**0.79**	**0.76**	**0.79**	− 0.30	**0.84**	**0.82**	0.17	**0.53**	**0.91**	La
Ce	**0.72**	**0.46**	**0.72**	0.33	0.11	**0.64**	**0.79**	**0.75**	**0.82**	− 0.22	**0.82**	**0.83**	0.31	**0.44**	**0.89**	Ce
Hf	**0.87**	**0.56**	**0.88**	**0.54**	0.23	**0.74**	**0.95**	**0.92**	**0.92**	− 0.26	**0.96**	**0.94**	0.10	**0.61**	**0.77**	Hf
Pb	− 0.09	− 0.02	0.00	0.39	0.09	0.37	− 0.04	− 0.03	− 0.03	− 0.43	0.04	− 0.02	0.11	0.36	0.28	Pb
Th	**0.65**	0.34	**0.72**	0.36	0.05	**0.59**	**0.76**	**0.73**	**0.79**	− 0.27	**0.81**	**0.79**	0.19	**0.57**	**0.84**	Th
U	**0.66**	0.41	**0.73**	0.28	0.07	**0.60**	**0.76**	**0.74**	**0.76**	− 0.21	**0.80**	**0.79**	0.26	**0.50**	**0.86**	U
	Na	Mg	Al	Cl	K	Ca	Sc	V	Cr	Mn	Fe	Co	Cu	Zn	As	

	Br	Rb	Sr	Mo	Cd	Sb	Cs	Ba	La	Ce	Hf	Pb	Th	U	
Na	r	r	p	p	r	r	r	p	r	r	r	r	r	r	Na
Mg	r	r	r	r	r	r	r	r	r	r	r	r	r	r	Mg
Al	r	r	r	r	r	p	r	p	p	p	p	r	p	p	Al
Cl	p	r	r	r	p	r	r	r	r	r	r	p	r	r	Cl
K	r	r	r	r	r	r	r	r	r	r	r	r	r	r	K
Ca	r	r	r	r	p	r	r	p	p	r	p	p	r	r	Ca
Sc	r	r	p	p	r	p	r	p	p	p	p	r	p	p	Sc
V	r	r	r	r	r	p	r	p	p	p	p	r	p	p	V
Cr	r	r	r	p	r	r	r	p	p	p	p	r	p	p	Cr
Mn	r	r	r	n	n	r	r	n	r	r	r	n	r	r	Mn
Fe	p	r	p	p	p	p	r	p	p	p	p	r	p	p	Fe
Co	p	r	p	p	r	p	r	p	p	p	p	r	p	p	Co
Cu	r	r	p	p	r	r	r	p	r	r	r	r	r	r	Cu
Zn	r	r	p	r	p	r	r	p	r	r	p	r	p	r	Zn
As	p	r	p	p	p	p	r	p	p	p	p	p	p	p	As
Br		n	r	r	r	p	r	r	p	p	r	p	p	r	Br
Rb	− 0.33		r	r	n	n	p	r	r	r	r	n	r	r	Rb
Sr	**0.54**	0.15		p	r	r	r	p	r	p	r	r	r	r	Sr
Mo	**0.42**	0.12	**0.62**		r	r	r	p	p	p	r	r	p	p	Mo
Cd	0.30	− 0.42	0.04	0.25		p	r	p	p	r	r	p	p	r	Cd
Sb	0.36	− **0.59**	0.12	0.14	0.38		r	r	p	p	p	p	p	p	Sb
Cs	− 0.08	**0.60**	0.17	0.04	− 0.27	− 0.24		r	r	r	r	r	r	r	Cs
Ba	**0.49**	0.12	**0.75**	**0.85**	0.25	0.25	0.03		p	p	p	r	p	p	Ba
La	**0.47**	− 0.24	0.40	**0.73**	0.34	**0.57**	− 0.18	**0.79**		p	p	p	p	p	La
Ce	**0.48**	− 0.10	**0.45**	**0.76**	0.16	**0.43**	− 0.12	**0.85**	**0.94**		p	r	p	p	Ce
Hf	0.13	0.03	0.39	**0.63**	0.12	0.34	− 0.04	**0.71**	**0.81**	**0.75**		r	p	p	Hf
Pb	0.36	− 0.48	0.06	0.20	**0.96**	**0.42**	− 0.24	0.20	0.26	0.08	0.02		r	r	Pb
Th	0.41	− 0.02	**0.42**	**0.72**	0.22	**0.51**	− 0.11	**0.81**	**0.90**	**0.92**	**0.80**	0.15		p	Th
U	0.38	− 0.06	**0.43**	**0.74**	0.18	**0.46**	− 0.17	**0.82**	**0.87**	**0.92**	**0.76**	0.10	**0.94**		U
	Br	Rb	Sr	Mo	Cd	Sb	Cs	Ba	La	Ce	Hf	Pb	Th	U	

Negative covariabilities (increase–decrease) in concentrations were also observed, in most cases for pairs of elements where one of the components was Mn, Rb, or Cd. In this study, manganese in a pair with Zn, Mo, Ba, Pb, and Cd appears to be a potent antagonist of these elements. This supports the thesis by Varela et al. (2015) that the concentrations of the elements can be attributed to causes other than the inherent physicochemical characteristics of the moss. Antagonisms and interactions among the some of these elements (Mo, Zn) and their effects on higher plant growth have been reported by a number of investigators (2011). Mn is the one of elements for which there was no significant correlation between the concentrations in moss and in the bulk deposition (Boquete et al. 2011). The relationship between exposure and concentration is altered by some as yet unknown reason (i.e., the physicochemical characteristics of the emissions, physicochemical and biological processes, and their interactions with environmental factors) (Boquete et al. 2011). Mn is an essential element for moss and, therefore, moss has a high capacity to regulate the levels of this element, unlike those of other contaminants, which may explain the dependence of elements considered to be derived primarily from anthropogenic sources (e.g., Pb and Cd). Although, at high concentrations Mn also may cause toxic effects (El-Joual and Cox 1998). For the alkali metal Rb, we observed negative covariabilities with Cd and Pb. Most probably, Rb can replace Cd and Pb cations in the exchange centres of mosses. For Rb there also were negative covariabilities with Br and Sb.

In most cases, the lowest *t* value in the complete data (positive covariabilities, increase- increase) was found for pairs of elements released into the environment primarily from anthropogenic (e.g., for pairs of elements regarded as anthropogenic in origin, mostly Pb–Cd, Zn–Cd, As–Sb) and crustal (e.g., for pairs of elements regarded as crustal in origin, mostly Al–V, Sc–Fe, Fe–Co, Th–U) sources. Low values of *t* assume proportionality of the listed element concentrations, assuming that in moss their constant ratio is required. The statistical methods used in this study can be useful in similar biomonitoring projects, especially in regional comparisons of mosses concentrations of trace elements.

## Conclusions

Moss samples were collected in unpolluted areas of national parks. The distribution of element concentrations was differentiated. Some of the distributions were significantly skewed and in the data outliers are expected. Although their elemental composition was not uniform, clear spatial patterns in concentration distributions were not observed. In the linear covariability estimation, the *t* quantile approach was used. A number of positive covariabilities were observed. This could be a result of the geochemical characteristics of the local environment, including the crust composition to which soil composition is related.

Due to the protection of the national park areas, the influence of pollution sources on the elemental composition of moss is limited. The concentrations of most elements (As, Ba, Ca, Cd, Cl, Cr, Cu, Mo, Pb, Sr, and Zn) were higher in mosses collected from highland and mountainous parks than in lowland parks, with the opposite relationship only for Mn. A comparison of data obtained from Polish national parks in 1970s and 1990s showed a significant decrease in the concentrations of heavy metals.

## Electronic supplementary material

Below is the link to the electronic supplementary material.

Supplementary file1 (DOC 76 kb)
