# Supplementary material for: The Moss Biomonitoring Method and Neutron Activation Analysis in Assessing Pollution by Trace Elements in Selected Polish National Parks
Source: Arch Environ Contam Toxicol. 2020 Sep 8;79(3):310–20. doi: 10.1007/s00244-020-00755-6 (PMC7578132; doi:10.1007/s00244-020-00755-6)
Supplement: Supplementary file 1 — Supplementary file1 (DOC 76 kb) [file 244_2020_755_MOESM1_ESM.doc]

**Supplementary Information**

Table A. Comparison of measured and certified concentration values (mg/kg) of apple leaves and Montana soil reference material (NIST, SRM-1515, 2711) for the neutron activation analysis (uncertainty in parentheses in %).

| Elements | Certified value | | Present work | |
| --- | --- | --- | --- | --- |
|  | Apple leaves | Montana soil | Apple leaves | Montana soil |
| As | 0.04 (18.4) | 105 (7.6) | 0.04 (15) | 62.6 (10) |
| Ba | 49 (4.1) | 726 (5.2) | 52.3 (3.5) | 766 (5) |
| Br | 1.8 (30) | 5 (30) | 1.77 (30) | 6.1 (30) |
| Ca | 15260 (1) | 28800 (2.8) | 15300 (1.2) | 16100 (3) |
| Ce | 3 (30) | 69 (30) | 2.87 (30) | 69 (30) |
| Co | 0.09 (30) | 10 (30) | 0.09 (30) | 12.2 (30) |
| Cr | 0.3 (30) | 47 (30) | 0.7 (30) | 59.9 (30) |
| Fe | 83 (6) | 28900 (2.1) | 82.3 (5.7) | 27000 (1.9) |
| K | 16100 (1.2) | 24500 (3.3) | 16200 (1.5) | 24400 (1.5) |
| La | 20 (30) | 40 (30) | 19.1 (30) | 37.9 (30) |
| Mg | 2710 (3) | 10500 (2.9) | 1050 (3.5) | 9978 (3.5) |
| Mn | 54 (5.6) | 638 (4.4) | 48.5 (7.2) | 645 (3.3) |
| Mo | 0.09 (13.8) | 1.6 (30) | 0.087 (15) | 1.6 (30) |
| Na | 24.4 (5) | 11400 (2.6) | 40 (6.1) | 9680 (3) |
| Rb | 10.2 (14.7) | 110 (30) | 10.5 (13.5) | 111 (30) |
| Sb | 0.013 (30) | 19.4 (9.3) | 0.014 (30) | 17.6 (10.1) |
| Sc | 0.03 (30) | 9 (30) | 0.029 (30) | 9.8 (25) |
| Sr | 25 (8) | 245.3 (0.3) | 23.7 (10) | 247 (0.5) |
| Th | 0.03 (30) | 14 (30) | 0.03 (30) | 13.5 (30) |
| U | 0.006 (30) | 2.6 (30) | 0.0056 (30) | 2.7 (30) |
| V | 0.26 (11.5) | 81.6 (3.6) | 0.22 (10) | 103 (3.7) |
| Zn | 12.5 (2.4) | 350.4 (1.4) | 11.7 (2) | 349 (1.5) |

Table B. Analysis of certified reference material for atomic absorption analysis.

| Element | Moss M2 (Finnish Forest Research Institute) standards | | | | Moss M3 (Finnish Forest Research Institute) standards | | | |
| --- | --- | --- | --- | --- | --- | --- | --- | --- |
|  | Certified  (mg kg^−^1) | Found | Recovery (%) | CV | Certified  (mg kg^−^1) | Found | Recovery (%) | CV |
| Cd | 0.454±0.019 | 0.460±0.013 | 101.32 | 2.8 | 0.106±0.005 | 0.109±0.004 | 102.83 | 3.7 |
| Cu | 67.7±2.5 | 68.2±1.4 | 100.74 | 2.1 | 3.76±0.23 | 3.80±0.10 | 101.06 | 2.8 |
| Pb | 6.37±0.43 | 6.31±0.22 | 99.06 | 3.6 | 3.33±0.25 | 3.29±0.10 | 98.79 | 3.2 |

Table C. Total emissions of heavy metals in Poland (in megagrams) (GUS 2016).

| element | 2000 | 2005 | 2010 |
| --- | --- | --- | --- |
| As | 39.7 | 45.4 | 43.9 |
| Cr | 46.9 | 43.3 | 44.8 |
| Zn | 1247.6 | 1296.7 | 1366.5 |
| Cd | 18.8 | 14.8 | 13.9 |
| Cu | 305.7 | 342.3 | 326.4 |
| Ni | 164.9 | 165 | 138.8 |
| Pb | 485.2 | 497.7 | 517.3 |
| Hg | 10.5 | 9.8 | 9.6 |

Table D. Statistical parameters of the element concentrations *c* (mg kg^−1^) in moss samples (*min* – minimal value, *q*_1_– lower quartile, median, arithmetic mean, *q*_3_– upper quartile, *max* – maximal value, SD – standard deviation, and MADN – normalized median absolute deviation about the median).

| *c* (mg kg^−1^) | *min* | *q*_1_ | median | mean | *q*_3_ | *max* | SD | MADN |
| --- | --- | --- | --- | --- | --- | --- | --- | --- |
| Al | 105 | 380 | 584 | 877 | 1125 | 3770 | 865 | 434 |
| As | 0.09 | 0.18 | 0.30 | 0.30 | 0.30 | 1.0 | 0.2 | 0.01 |
| Ba | 4.0 | 11.3 | 14.5 | 20.5 | 20.8 | 78 | 17 | 5.9 |
| Br | 0.8 | 1.4 | 1.9 | 2.0 | 2.5 | 5.2 | 1.0 | 0.9 |
| Ca | 740 | 2082 | 2750 | 3086 | 3557 | 9030 | 1920 | 1119 |
| Cd | 0.20 | 0.25 | 0.30 | 0.38 | 0.42 | 1.10 | 0.21 | 0.11 |
| Ce | 0.23 | 0.44 | 0.71 | 1.00 | 1.10 | 3.90 | 0.94 | 0.41 |
| Cl | 39 | 97 | 173 | 236 | 293 | 813 | 193 | 135 |
| Co | 0.1 | 0.2 | 0.2 | 0.4 | 0.3 | 2.3 | 0.5 | 0.1 |
| Cr | 0.4 | 1.4 | 2.0 | 2.9 | 2.5 | 16.8 | 3.7 | 0.8 |
| Cs | 0.04 | 0.22 | 0.26 | 0.41 | 0.42 | 2.2 | 0.44 | 0.18 |
| Cu | 4.9 | 5.3 | 7.6 | 7.5 | 9.3 | 10.8 | 1.9 | 2.9 |
| Fe | 119 | 243 | 403 | 665 | 588 | 4600 | 964 | 274 |
| Hf | 0.02 | 0.06 | 0.09 | 0.15 | 0.18 | 0.86 | 0.17 | 0.07 |
| K | 2600 | 5070 | 6425 | 6787 | 7558 | 14900 | 2812 | 1957 |
| La | 0.10 | 0.23 | 0.40 | 0.53 | 0.60 | 1.90 | 0.43 | 0.30 |
| Mg | 454 | 915 | 1275 | 1912 | 2595 | 6320 | 1534 | 606 |
| Mn | 28 | 127 | 270 | 313 | 518 | 671 | 206 | 259 |
| Mo | 0.07 | 0.11 | 0.20 | 0.20 | 0.20 | 0.40 | 0.08 | 0.07 |
| Na | 98.7 | 127 | 159 | 254 | 239 | 1450 | 291 | 66.7 |
| Pb | 3.1 | 4.9 | 6.1 | 7.6 | 8.2 | 22.3 | 4.8 | 2.7 |
| Rb | 6 | 24 | 30 | 36 | 47 | 100 | 24 | 25 |
| Sb | 0.05 | 0.14 | 0.19 | 0.18 | 0.24 | 0.31 | 0.07 | 0.07 |
| Sc | 0.03 | 0.06 | 0.12 | 0.25 | 0.18 | 0.24 | 0.52 | 0.08 |
| Sr | 2.6 | 5.9 | 8.6 | 10.6 | 11.1 | 34.9 | 7.8 | 4 |
| Th | 0.03 | 0.06 | 0.1 | 0.13 | 0.17 | 0.44 | 0.09 | 0.07 |
| U | 0.02 | 0.03 | 0.04 | 0.05 | 0.06 | 0.18 | 0.03 | 0.03 |
| V | 0.3 | 0.8 | 1.2 | 1.8 | 2 | 10.1 | 2.2 | 0.7 |
| Zn | 12 | 39 | 44.5 | 46.7 | 52.5 | 83 | 13.8 | 8.9 |
